# Electron impact ionisation cross sections of *cis*- and *trans*-diamminedichloridoplatinum(II) and its hydrolysis products

**DOI:** 10.1080/00268976.2018.1509148

**Published:** 2018-08-14

**Authors:** Stefan E. Huber, Daniel Süß, Michael Probst, Andreas Mauracher

**Affiliations:** Institute of Ion Physics and Applied Physics, Leopold-Franzens-University Innsbruck, Innsbruck, Austria

**Keywords:** Electron-impact ionisation, total cross sections, cisplatin, transplatin, hydrolysis

## Abstract

We report total electron-impact ionisation cross sections (EICSs) of cisplatin, its hydrolysis products and transplatin in the energy range from threshold to 10 keV using the binary-encounter-Bethe (BEB) and its relativistic variant (RBEB), and the Deutsch-Märk (DM) methods. We find reasonable agreement between all three methods, and we also note that the RBEB and the BEB methods yield very similar (almost identical) results in the considered energy range. For cisplatin, the resulting EICSs yield cross section maxima of 22.09 × 10^−20^ m^2^ at 55.4 eV for the DM method and 18.67 × 10^−20^ m^2^ at 79.2 eV for the (R)BEB method(s). The EICSs of monoaquated cisplatin yield maxima of 12.54 × 10^−20^ m^2^ at 82.8 eV for the DM method and of 9.74 × 10^−20^ m^2^ at 106 eV for the (R)BEB method(s), diaquated cisplatin yields maxima of 7.56 × 10^−20^ m^2^ at 118.5 eV for the DM method and of 5.77 × 10^−20^ m^2^ at 136 eV for the (R)BEB method(s). Molecular geometry does not affect the resulting EICS significantly, which is also reflected in very similar EICSs of the *cis*- and *trans*-isomer. Limitations of the work as well as desirable future directions in the research area are discussed.

## GRAPHICAL ABSTRACT

**Figure:**
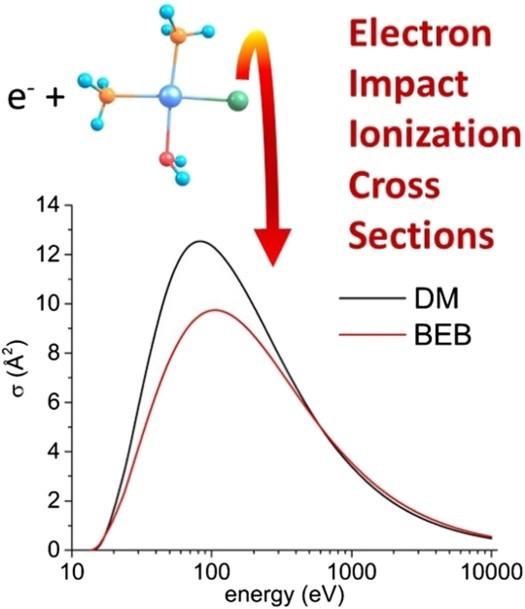

## 1. Introduction

**Figure 1.**
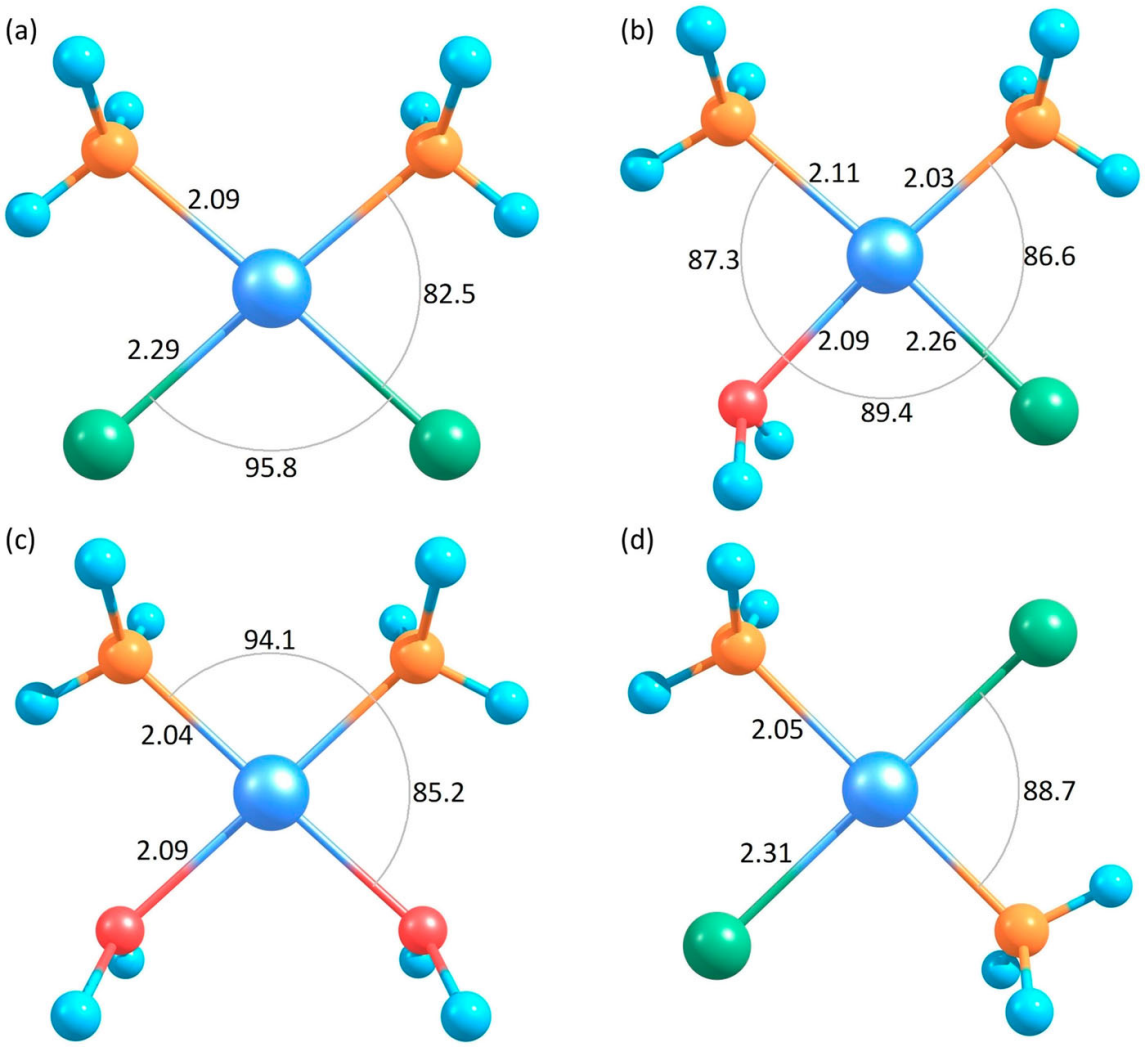
Optimised structures of (a) cisplatin, (b) mono-aquated cisplatin, i.e. *cis*-[Pt(NH_3_)_2_(OH_2_)Cl]^+^, (c) di-aquated cisplatin, i.e. *cis*-[Pt(NH_3_)_2_(OH_2_)_2_]^2+^ and (d) transplatin. Hydrogen, nitrogen, oxygen, chloride and platinum atoms are depicted as light blue, orange, red, green and metallic blue, respectively. Bond lengths are given in Å (= 10^-10^ m), bond angles in degrees. (Colour online, B&W in print)

Having been discovered as early as 1845, *cis*-diamminedichloridoplatinum(II), cis-Pt(NH_3_)_2_Cl_2_, in short, cisplatin, see Figure 1(a), is up to now one of the most leading drugs used in anticancer chemotherapy. Subsequently to the administration of the drug and its transfer into cells, the Pt-Cl bonds are hydrolised [1-3], i.e. the chloride ligands are replaced by water molecules. Replacement of one ligand results in monoaquated cisplatin, i.e. *cis*-[Pt(NH_3_)_2_(OH_2_)Cl]^+^, replacement of both ligands results in diaquated cisplatin, i.e. *cis*-[Pt(NH_3_)_2_(OH_2_)_2_]^2+^ see Figure 1(b,c), respectively. The hydrolysed products then bind to DNA, forming intra-strand cross-links between nucleobases. This inhibits the cell replication process, which is the primary mode of action of the drug’s anticancer activity [1-4]. Most interestingly, the isomer transplatin, see Figure 1(d), is clinically inactive [5].

Chemotherapy is often combined with radiotherapy in order to mutually enhance the effectiveness of the two treatments. It is well known that cisplatin acts as a radiosensitiser, i.e. it substantially enhances DNA damage and tumour cell killing rates upon irradiation of the targeted cells [6-11]. In case of the latter, products of ionising radiation such as electrons not only interact with the biomolecular environment but also with the administered drugs. Electron-impact ionisation processes are among the dominant processes for electron-molecule scattering phenomena playing a role also in interatomic Coulombic decay (ICD) driven by energy transfer [12]. Here, ICD describes relaxation of an ionised compound by transferring its excess energy to a neighbouring molecule, which becomes also ionised by this process, leading to two positively charged products that repel each other and subsequently often break apart [13]. Hence, ICD is one of the processes that needs to be considered especially in contexts of electron interaction with molecules in biological environments. Data on such processes and particularly on the probability distribution characterising the interaction of the ionising radiation with the cell as a function of impact energy are required as input for modelling purposes using e.g. Monte-Carlo track structure simulations [14,15]. The distribution of the energies of secondary electrons, i.e. electrons produced by highly-energetic primary ionising radiation, has its maximum typically in the range of a few tens of eV (= 1.602 × 10^−19^ J) beyond which it decreases up to primary impact energies of about 10 keV [16]. This decrease may be described by a power law. For even higher energies the distribution falls off more rapidly to virtually zero [16].

In this contribution, we report calculated electron-impact ionisation cross sections (EICSs) for cisplatin and its hydrolysis products as well as for transplatin from threshold to 10 keV. We compare two of the most-widely used methods for the computation of EICSs, i.e. binary-encounter-Bethe (BEB) theory of Kim et al. [17,18] and the Deutsch-Märk (DM) method [19]. Both types of methods have been successfully applied to atoms, molecules, clusters, ions and radicals. Their accuracy is typically in the same range as the one of experimental data [20].

EICSs for cisplatin have been reported earlier using also the BEB approach [21] and employing the multi-scattering centre spherical complex optical potential approach [22]. However, cisplatin’s modes of anticancer action are based on the formation of its hydrolysis products in target cells [1-3,23]. Hence, in order to model the interaction of radiation with cells it is important to know EICSs for mono- and diaquated cisplatin which we report here. In addition, we compare the EICSs obtained for cisplatin with those obtained for its clinically inactive isomer transplatin. Moreover, we assess some methodological issues including the importance of the usage of effective core potentials for determination EICSs using the BEB method as well as the magnitude of relativistic corrections especially at elevated impact energies when dealing with heavy elements such as Pt.

## 2. Methods

In this section, we provide a short overview of the DM method in Section 2.1, the BEB method in Section 2.2 and details of the quantum chemical calculations used to obtain the required input for those methods in Section 2.3.

### 2.1 The Deutsch-Märk (DM) method

The DM method was originally developed as an easy-to-use, semi-empirical approach for the calculation of EICSs of atoms in their electronic ground state from threshold to about 100 eV [19]. In a more recent variant of the DM method [20,24], the total single electron-impact ionisation cross section σ of an atom is expressed as:

(1) $$\sigma_{DM}(u)=\underset{n,l}{\sum }g_{nl}\pi r_{nl}^{2}\xi_{nl}b_{nl}^{\left(q\right)}(u)[\text{ln}(c_{nl}u)/u],$$

where $r_{nl}$ is the radius of maximum radial density of the atomic sub-shell characterised by quantum numbers n and l (as listed in column 1 in the tables of Desclaux [25]) and $\xi_{nl}$ is the number of electrons in that sub-shell. The sum extends over all atomic sub-shells labelled by n and l. The $g_{nl}$ are weighting factors, which were originally determined by a fitting procedure [26,27] using reliable experimental cross section data for a few selected atoms, for which the accuracy of the reported rate is in the range of 7–15%. The reduced energy u is given by $u=E/E_{nl}$, where E refers to the incident energy of the electrons and $E_{nl}$ denotes the ionisation energy of the sub-shell characterised by n and l. The energy-dependent quantities $b_{nl}^{\left(q\right)}(u)$ were introduced in an effort to merge the high-energy region of the ionisation cross section, which follows the Born-Bethe approximation [28], with the DM formula of the cross sections in the regime of low impact energies. The function $b_{nl}^{\left(q\right)}$ in Equation (1) has the explicit form:

(2) $$b_{nl}^{\left(q\right)}=\frac{A_{1}-A_{2}}{1+{\left(u/A_{3}\right)}^{p}}+A_{2}.$$

The four constants $A_{1}$, $A_{2}$, $A_{3}$ and p were determined, together with $c_{nl}$, from reliably measured cross sections for the various values of n and l. The superscript q refers to the number of electrons in the (n,l)-th sub-shell and allows the possibility to use slightly different functions $b_{nl}^{\left(q\right)}$ depending on the number of electrons in the respective sub-shell. At high impact energies u goes to infinity, the first term in Equation (2) goes to zero and $b_{nl}^{\left(q\right)}(u)$ becomes a constant ensuring the high-energy dependence of the cross sections predicted by the Born-Bethe theory [28].

The DM formalism has been extended to the calculation of EICSs of atoms in excited states, molecules and free radicals, atomic and molecular ions, and clusters [20]. For the calculation of the EICS of a molecule, a population analysis [29,30] must be carried out to obtain the weights with which the atomic orbitals of the constituent atoms contribute to each occupied molecular orbital. These weights are obtained from the coefficients of the occupied molecular orbital after a transformation employing the overlap matrix in order to correct for the non-orthogonality of the atomic basis functions.

### 2.2 The binary-encounter-Bethe (BEB) method

The BEB model [18] was derived from the binary-encounter-dipole model [17] by replacing the df/dE term for the continuum dipole oscillator strengths by a simpler form. Thus, a modified form of the Mott cross section together with the asymptotic form of the Bethe theory describing the electron-impact ionisation of an atom was combined into an expression for the cross section of each molecular orbital:

(3) $$\begin{array}{rl} \sigma_{BEB}(t) & =\frac{S}{t+u+1}\\ & \;\times \left[\frac{\text{ln}(t)}{2}\left(1-\frac{1}{t^{2}}\right)+1-\frac{1}{t}-\frac{\text{ln}(t)}{t+1}\right], \end{array}$$

where t=T/B, u=U/B, $S=4\pi a_{0}^{2}NR^{2}/B^{2}$, $a_{0}$ denotes the Bohr radius (0.5292 Å), R is the Rydberg energy (13.6057 eV), and T denotes the incident electron energy. N, B and U are the electron occupation number, the binding energy (ionisation energy), and the average kinetic energy of the respective molecular orbital, respectively. In the BEB model, the total cross section, similarly to the DM method, is then obtained by summation over the cross sections for all molecular orbitals.

The quantum chemical data needed to calculate EICSs are normally derived from all-electron calculations. For heavy elements and molecules containing them valence-shell-only calculations using effective-core potentials (ECPs) [31] can be used. This facilitates the quantum chemical calculations and allows, to some extent, the incorporation of relativistic effects stemming from inner electrons with high kinetic energy. Due to the lack of inner radial nodes of the pseudo-valence orbitals, their kinetic energies are lower than normal and Equation (3) can be used to determine the BEB cross section [32]. Using the BEB method in conjunction with ECPs has earlier been recommended over the use of all-electron basis sets for molecules that contain heavy (with atomic number *Z* > 10) atoms [33]. Moreover, in an earlier work on iron hydrogen clusters, a better agreement between EICSs obtained with BEB using ECPs and DM cross sections than between all-electron BEB and DM cross sections was found [34].

The cross section formula given by Equation (3) has experienced several modifications over the years and has been extended also to relativistic incident energies [35]. For the latter case the expression for the cross section reads:

(4) $$\begin{array}{rl} \sigma_{RBEB} & =\frac{4\pi a_{0}^{2}\alpha^{4}N}{(\beta_{t}^{2}+\beta_{u}^{2}+\beta_{b}^{2})2b^{\prime }}\\ & \;\times \left\{\frac{1}{2}\left[\ln\left(\frac{\beta_{t}^{2}}{1-\beta_{t}^{2}}\right)-\beta_{t}^{2}-\ln(2b^{\prime })\right]\right.\\ & \;\times \left.\left(1-\frac{1}{t^{2}}\right)+1-\frac{1}{t}-\frac{\ln t}{t+1}\frac{1+2t^{\prime }}{{\left(1+t^{\prime }/2\right)}^{2}}\right.\\ & \;\times \left.+\frac{b^{\prime 2}}{(1+t^{\prime }/2{)}^{2}}\frac{t-1}{2}\right\}, \end{array}$$

where α denotes the fine structure constant, $\beta_{t}^{2}=1-\frac{1}{{\left(1+t^{\prime }\right)}^{2}}$, $\beta_{b}^{2}=1-\frac{1}{{\left(1+b^{\prime }\right)}^{2}}$, $\beta_{u}^{2}=1-\frac{1}{{\left(1+u^{\prime }\right)}^{2}}$, $t^{\prime }=T/mc^{2}$, $b^{\prime }=B/mc^{2}$, $u^{\prime }=U/mc^{2}$ and c is the speed of light.

For the purpose of comparison we also used Equation (4) to compute EICSs for the molecules under consideration. We refer to those cross sections using simply the abbreviation RBEB.

### 2.3 Quantum chemical calculations

Molecular geometries were optimised using the TPSSh [36-38] density functional in conjunction with the Def2-TZVP basis set [39,40]. Relaxed structures and some structural parameters are depicted in Figure 1. The orbital populations required for the DM formalism were subsequently determined via Hartree–Fock (HF) calculations in conjunction with the minimal CEP-4G basis set [41-43]. Occupation, binding energy and average kinetic energy for each molecular orbital as required for the calculation of the BEB cross sections were also calculated at the HF/Def2-TZVP level of theory. Orbital energies for the outermost valence electrons were refined using results obtained with Outer-Valence-Green’s-Function (OVGF) electron-propagator theory [44] in conjunction with the Def2-TZVP basis set. Generally, split-valence triple-zeta basis sets with additional polarisation functions such as 6-311G(2df,2p) have been recommended for this kind of calculations [45]. In order to explore the reliability of the basis set employed by us, we computed vertical ionisation energies (VIEs) of the several constituents of the molecules under consideration, i.e. H_2_O, NH_3_, Cl and Pt, employing various basis sets. In Table 1, we provide the resulting VIEs together with experimental values. In line with earlier work on various closed-shell molecules [46], we observe that the results obtained with double-zeta basis sets can deviate substantially from both experimental values as well as from results obtained with triple-zeta basis sets with additional polarisation functions. Moreover, results obtained with the 6-311G(2df,2p) and the Def2-TZVP basis sets are in good agreement with each other and also closest to the available experimental values. Hence, we assume that the used level of theory yields reasonable electron binding energies also for trans- and cisplatin and its hydrolysis products. All quantum chemical calculations were performed with the Gaussian 16 software [47].

**Table 1:** Vertical ionisation energies (in eV) for the constituents of the molecules under consideration as obtained with the OVGF method using different basis sets.

Ion	6-31G(d,p)	6-311G(d,p)	6-311G(2df,2p)	Def2-SVP	Def2-TZVP	Exp.
H_2_O(1b_1_)	18.29 (−0.43)	18.31 (−0.41)	18.60 (−0.12)	18.27 (−0.45)	18.52 (−0.20)	18.72 [48]
H_2_O(3a_1_)	14.83 (−0.23)	14.56 (−0.27)	14.79 (−0.04)	14.48 (−0.35)	14.96 (0.13)	14.83 [48]
H_2_O(1b_2_)	12.21 (−0.57)	12.22 (−0.57)	12.47 (−0.31)	12.18 (−0.60)	12.64 (−0.14)	12.78 [48]
NH_3_(3a_1_)	15.96 (−0.84)	16.01 (−0.79)	16.21 (−0.59)	15.97 (−0.83)	16.29 (−0.51)	16.80 [48]
NH_3_(3a_1_)	10.50 (−0.30)	10.60 (−0.20)	10.80 (−0.01)	10.47 (−0.33)	10.86 (0.06)	10.80 [48]
Cl(3p)	12.31 (−0.66)	12.38 (−0.59)	12.78 (−0.19)	12.28 (−0.69)	12.74 (−0.23)	12.97 [49]
Pt(6s)	n.a.	n.a.	n.a.	8.86 (0.04)	8.85 (0.03)	8.82 [50]

## 3. Results and discussion

**Figure 2.**
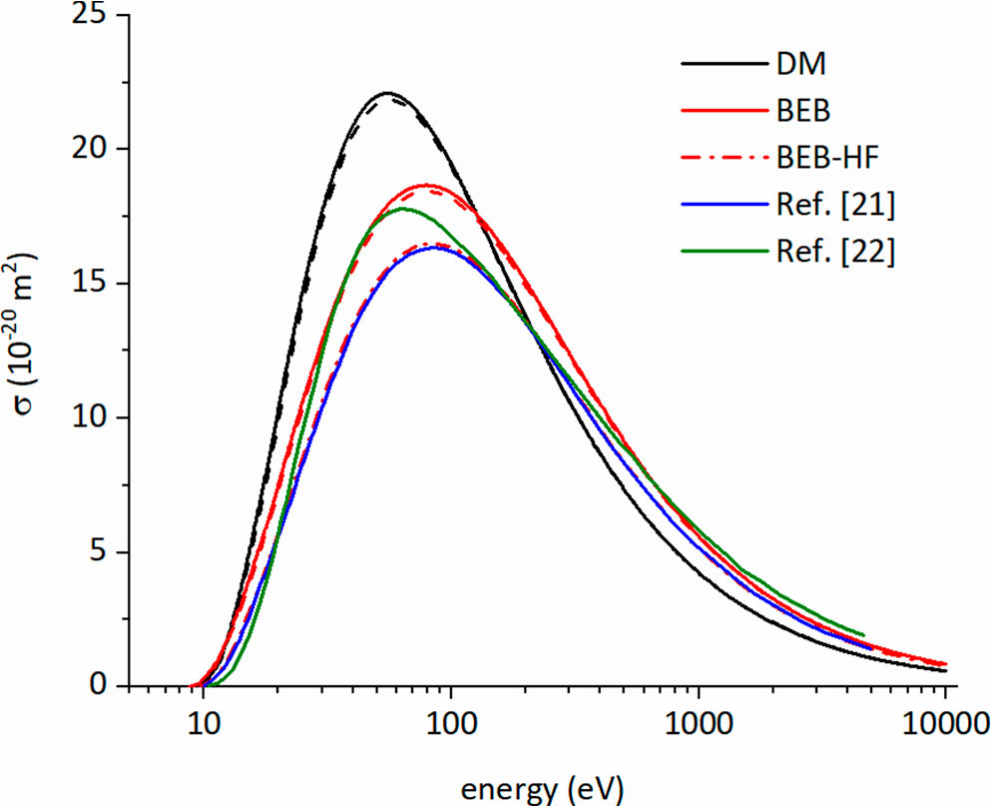
Total EICSs for cisplatin (solid lines) and transplatin (dashed lines) obtained with the DM (black line) and BEB (red line) methods. The cross sections obtained by Żywicka and Możejko [21] (blue line) and by Mahato et al. [22] (green line) are also depicted for comparison. Moreover, we depict also the BEB cross section which results if the HF orbital binding energies are used instead of the refined ones using the OVGF method (red dash-dotted line). (Colour online, B&W in print)

The EICSs of cis- and transplatin, as obtained with the various methods described above, are depicted in Figure 2. We also depict the results reported earlier by Żywicka and Możejko [21] and by Mahato et al. [22]. We find that both methods, i.e. DM and BEB, are in reasonable agreement with each other, deviating by about 15–20% (at energies of the cross section maxima) from each other. The relativistic BEB method, i.e. RBEB, yields EICSs for cis- and transplatin at the considered impact energies that differ only negligibly from the standard BEB ones. These differences increase monotonically with increasing impact energy and become maximal for the highest considered impact energy of 10 keV. For instance, for cisplatin the maximum difference is as small as 0.02 × 10^−20^ m^2^ at 10 keV which corresponds to a deviation of about 3% between BEB and RBEB at this energy. Graphically, the differences between the respective curves are not discernible, so the RBEB EICSs are not explicitly included in Figure 2. The differences between the EICSs of the two considered isomers are also very small. This is in line with the earlier finding, in a work on beryllium tungsten clusters [51], that differences in molecular geometries and even the differences between isomers do not affect the resulting EICSs substantially. In particular, we obtain cross section maxima of 22.09 × 10^−20^ m^2^ at 55.4 eV for the DM method and 18.67 × 10^−20^ m^2^ at 79.2 eV for the BEB and RBEB methods for cisplatin, and of 21.85 × 10^−20^ m^2^ at 56.2 eV for the DM method and 18.44 × 10^−20^ m^2^ at 79.6 eV for the BEB and RBEB methods for transplatin. We note that Żywicka and Możejko [21] employed also the BEB method and obtained a somewhat lower EICS for cisplatin with a maximum of 16.31 × 10^−20^ m^2^ at 85 eV. This difference could stem from approximating the ionisation energies by HF calculations (Koopman’s theorem) instead of calculating them via the OVGF method; calculating a BEB EICS for cisplatin using the ionisation energies from HF calculations (Koopman’s theorem) as input (see the red, dash-dotted line in Figure 2) results in a cross section in very good agreement with the one of Żywicka and Możejko [21]. The remaining difference can be attributed to the different basis sets used in their and in our work. However, the difference between the BEB methods using HF and OVGF binding energies puts emphasis on how the accuracy of the method depends on the supplied orbital binding energies, especially the ones highest in energy (which contribute most to the EICS). Also the EICS reported by Mahato et al. [22] is slightly smaller than the BEB and DM EICSs reported here. In their work, the ionisation cross section was determined by (energy-dependent) scaling of the total inelastic cross section, comprising the sum of total excitation and total ionisation cross sections, computed using a multi-scattering centre spherical complex optical potential approach [22]. In the region of the maximum the scaling factor was chosen as 0.75 [22]. Hence, our results indicate that this scaling factor might be slightly too low for cisplatin.

**Figure 3.**
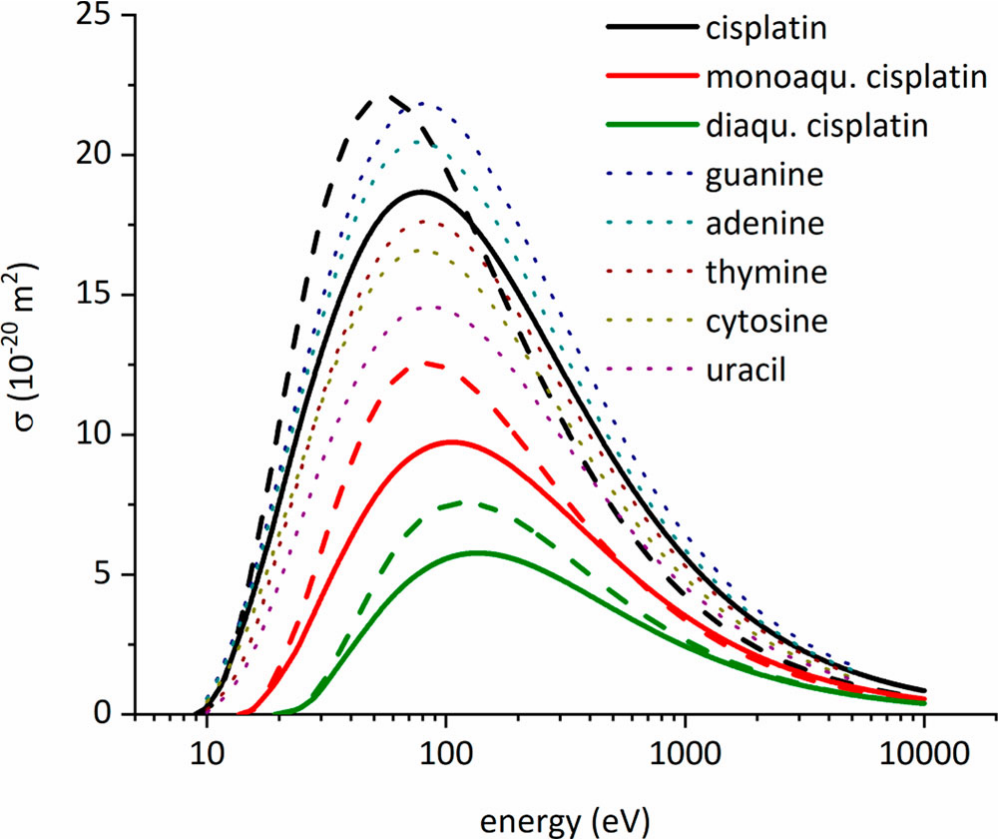
BEB (solid lines) and DM (dashed lines) EICSs for cisplatin (black lines), monoaquated cisplatin (red lines) and diaquated cisplatin (green lines). For comparison, also the total ionisation cross sections obtained by Możejko and Sanche [52] for DNA and RNA bases are included (dotted lines). (Colour online, B&W in print)

In Figure 3, we depict the obtained EICSs for cisplatin and its hydrolysis products together with earlier results obtained for the nucleobases adenine, cytosine, guanine, thymine and uracil [52]. Whereas Żywicka and Możejko [21] found that the EICS of cisplatin is comparable to the ones of the pyrimidine bases cytosine, thymine and uracil, but considerably lower than the ones for the purine bases adenine and guanine, our results indicate rather the opposite, see Figure 3. Moreover, as pointed out in the introduction, the cytotoxic effects of cisplatin require its hydrolysis which takes place upon uptake of the molecule into the cell and hence, from a pharmacological point of view, the EICSs of the respective hydrolysis products are even more important than the one of cisplatin per se. The EICSs of mono- and diaquated cisplatin, however, are clearly substantially lower than the ones of both pyrimidine and purine derivatives. The EICSs of monoaquated cisplatin yield maxima of 12.54 × 10^−20^ m^2^ at 82.8 eV for the DM method and of 9.74 × 10^−20^ m^2^ at 106 eV for the BEB and RBEB methods, the ones of diaquated cisplatin yield maxima of 7.56 × 10^−20^ m^2^ at 118.5 eV for the DM method and of 5.77 × 10^−20^ m^2^ at 136 eV for the BEB and RBEB methods.

Concerning the accuracy of the calculated cross sections we note the following. Differences between the DM and BEB (and also the RBEB) methods account for about 30% in the region around the cross section maxima for all molecules under consideration with the DM cross sections being generally larger than the BEB (and RBEB) ones. For increasing impact energies the DM cross sections decrease faster than the BEB cross sections. At 10 keV, the latter are about 45% larger than the DM cross sections for cis- and transplatin, whereas they are about 15% larger for monoaquated cisplatin and almost identical (less than 1% difference) for diaquated cisplatin. Close to the threshold energy the resulting cross sections can deviate more substantially from each other due to the different mathematical description of the near-threshold behaviour in the used models. Differences of the mentioned orders of magnitude between the two semi-empirical approaches are not uncommon [20,27]. In the absence of experimental data the accuracy of the calculated cross sections may very conservatively be estimated by the above stated ranges of deviations between the results obtained with the used semi-empirical approaches. Hence, we tentatively estimate the obtained cross sections to be accurate within at least 30% in an energy range of 20–100 eV (around the maxima) and at least within 45% for energies beyond 100 eV.

We would like to conclude this section with the discussion of two points concerning limitations of the presented work. Over the last decade, the original DM as well as the BEB methods experienced several modifications yielding improvements on resulting EICSs for atomic targets especially for relativistic impact energies, heavy elements in high charge states and effects related to inner shell ionisation [53-58]. Although the magnitude of corrections caused by these modifications is small in the energy range considered in this work, attempts to further adapt both approaches for molecular targets seem nevertheless desirable especially if impact energies in the range of and beyond several tens of keV become interesting.

An intrinsic limitation stems from the fact that all of the methods used in this work and discussed above yield total EICSs of the target molecules under consideration, i.e. sums of all partial cross sections characterising any reaction channel yielding at least one singly ionised molecular fragment (including the parent molecular ion) as a product. In particular, for a parent molecule AB, there exist three such reaction channels (regarding for convenience now only single ionisation events):

(4a) $${\text{e}}^{-}+\text{AB}\to \text{A}{\text{B}}^{+}+2{\text{e}}^{-}$$

(4b) $$\to \text{A}{\text{B}}^{+*}\to {\text{A}}^{+}+\text{B}+2{\text{e}}^{-}$$

(4c) $$\to \text{A}{\text{B}}^{+*}\to \text{A}+{\text{B}}^{+}+2{\text{e}}^{-}$$

Note that reactions (4b) and (4c) describe two-step dissociative ionisation mechanisms which proceed via intermediate formation of a transient excited positive ion.

Each of reactions (4a–c) is associated with its specific partial EICSs and the sum of all three yields the total EICS (for single ionisation) of the molecule AB. In order to inform modellers using e.g. Monte Carlo track structure simulations [14,15], knowledge of the partial EICSs for specific ionisation channels would be highly valuable. However, experimental as well as theoretical data on partial cross sections is very scarce. Although a purely theoretical approach based on first-principles for the prediction of partial EICSs is lacking, a few semi-empirical attempts have been made so far. Semi-empirical calculations have been used in conjunction with experimental data on oscillator strengths [59] or using experimental mass spectra [60,61] for the estimation of partial ionisation cross sections. A significant body of experimental data on partial and total EICSs of hydrocarbons was also assessed [62] revealing several empirical rules by which those cross sections are governed. It was found that (i) EICSs can be relatively well characterised by a cubic function of energy close to the associated reaction threshold and (ii) that the contribution of a specific reaction channel to the total cross section (i. e. the branching ratio) becomes asymptotically, approximately constant at elevated energies well beyond 20–30 eV [62], whereas the latter finding is a significantly better approximation (within 20%) for dominant reaction channels than for subdominant ones (for which variations of the branching ratio up to a factor of 6 were reported) [63]. We hold the view that further attempts how to make at least some empirically informed guesses on partial EICSs, possibly combining some of the attempts mentioned above, are most desirable in future work in this area besides highly needed experimental investigations.

## 4. Conclusion

We calculated total electron-impact ionisation cross sections (EICSs) of cisplatin, its hydrolysis products and transplatin in the energy range from threshold to 10 keV using binary-encounter-Bethe (BEB) and Deutsch-Märk (DM) methods. We find reasonable agreement between the two methods. For cisplatin, the resulting EICSs yield maxima of 22.09 × 10^−20^ m^2^ at 55.4 eV for the DM method and of 18.67 × 10^−20^ m^2^ at 79.2 eV for the BEB method. The EICSs of monoaquated cisplatin yield maxima of 12.54 × 10^−20^ m^2^ at 82.8 eV for the DM method and of 9.74 × 10^−20^ m^2^ at 106 eV for the BEB method, the ones of diaquated cisplatin yield maxima of 7.56 × 10^−20^ m^2^ at 118.5 eV for the DM method and of 5.77 × 10^−20^ m^2^ at 136 eV for the BEB method. We compared the results also with the relativistic variant of the BEB method (RBEB) which yields very similar results in the considered energy range. Also the EICS for transplatin is very similar to the one of cisplatin. Overall, we tentatively (and very conservatively) estimate the accuracies of the obtained cross sections to be within at least 30% in an energy range of 20–100 eV (around the maxima) and at least within 45% for energies beyond 100 eV. A comparison of the results with EICSs reported for pyrimidine and purine nucleobases yields comparable ionisation cross sections of cisplatin but substantially lower ones of its hydrolysis products which are actually responsible for cytotoxic activity of the drug in targeted cells.

## References

[1] Guo Z.J. and Sadler P.J., Angew Chem, Int Ed. 38, 1513 (1999). 10.1002/(SICI)1521-3773(19990601)38:11<1512::AID-ANIE1512>3.0.CO;2-Y 29711002

[2] Fuertes M.A., Castilla J., Alonso C. and Perez J.M., Curr. Med. Chem. 10, 257 (2003). doi: 10.2174/0929867033368484 12570712

[3] Fuertes M.A., Alonso C. and Perez J.M., Chem. Rev. 103, 645 (2003). doi: 10.1021/cr020010d 12630848

[4] Lau J.K.C. and Ensing B., Phys. Chem. Chem. Phys. 12, 10348 (2010). doi: 10.1039/b918301a 20582358

[5] Nafisi S. and Norouzi Z., DNA Cell Biol. 28, 469 (2009). doi: 10.1089/dna.2009.0894 19558218

[6] Rezaee M., Sanche L. and Hunting D.J., Radiat. Res. 179, 323 (2013). doi: 10.1667/RR3185.1 23368416

[7] Rezaee M., Hunting D.J. and Sanche L., Int J. Radiat. Oncol. Biol. Phys. 87, 847 (2013). doi: 10.1016/j.ijrobp.2013.06.2037 23910707 PMC3817081

[8] Behmand B., Wagner J.R., Sanche L. and Hunting D.J., J. Phys. Chem. B. 118, 4803 (2014). doi: 10.1021/jp5014913 24779712 PMC4623755

[9] Chen H.Y., Chen H.F., Kao C.L., Yang P.Y. and Hsu S.C.N., Phys. Chem. Chem. Phys. 16, 19290 (2014). doi: 10.1039/C4CP02306D 25098629

[10] Sahbani S.K., Rezaee M., Cloutier P., Sanche L. and Hunting D.J., Chem-Biol. Interact. 217, 9 (2014). doi: 10.1016/j.cbi.2014.04.004 24732435

[11] Behmand B., Marignier J.L., Mostafavi M., Wagner J.R., Hunting D.J. and Sanche L., J. Phys. Chem. B. 119, 9496 (2015). doi: 10.1021/acs.jpcb.5b01752 26098937 PMC4623752

[12] Ren X.G., Al Maalouf E.J., Dorn A. and Denifl S., Nat. Commun. 7 (6), 11093 (2016). doi: 10.1038/ncomms11093 27000407 PMC4804183

[13] Cederbaum L.S., Zobeley J. and Tarantelli F., Phys. Rev. Lett. 79, 4778 (1997). doi: 10.1103/PhysRevLett.79.4778

[14] Dingfelder M., Radiat. Prot. Dosim. 122, 16 (2006). doi: 10.1093/rpd/ncl494 17277326

[15] Müller M., Durante M., Stocker H., Merz F. and Bechmann I., Eur. Phys. J. D. 60, 171 (2010). doi: 10.1140/epjd/e2010-00030-y

[16] Pimblott S.M. and LaVerne J.A., Radiat. Phys. Chem. 76, 1244 (2007). doi: 10.1016/j.radphyschem.2007.02.012

[17] Kim Y.K. and Rudd M.E., Phys. Rev. A. 50, 3954 (1994). doi: 10.1103/PhysRevA.50.3954 9911367

[18] Kim Y.K., Ali M.A. and Rudd M.E., J. Res. Natl. Inst. Stand. Technol. 102, 693 (1997). doi: 10.6028/jres.102.046 27805116 PMC4894591

[19] Deutsch H. and Märk T.D., Int. J. Mass. Spectrom. 79, R1 (1987). doi: 10.1016/0168-1176(87)83009-4

[20] Deutsch H., Becker K., Probst M. and Märk T.D., in *Advances in Atomic, Molecular, and Optical Physics*, edited by Arimondo E., Berman P.R. and Lin C.C. (Elsevier Academic Press Inc, San Diego, 2009), Vol 57, p. 87.

[21] Żywicka B. and Możejko P., Eur. Phys. J. D. 66 (2), 54 (2012). doi: 10.1140/epjd/e2012-20697-0

[22] Mahato D., Naghma R., Alam M.J., Ahmad S. and Antony B., Mol. Phys. 114, 3104 (2016). doi: 10.1080/00268976.2016.1219408

[23] Jamieson E.R. and Lippard S.J., Chem. Rev. 99, 2467 (1999). doi: 10.1021/cr980421n 11749487

[24] Deutsch H., Scheier P., Becker K. and Märk T.D., Int. J. Mass. Spectrom. 233 (13), (2004).

[25] Desclaux J.P., At Data Nucl. Data Tables. 12, 311 (1973). doi: 10.1016/0092-640X(73)90020-X

[26] Margreiter D., Deutsch H. and Märk T.D., Int. J. Mass. Spectrom Ion Processes. 139, 127 (1994). doi: 10.1016/0168-1176(94)90024-8

[27] Deutsch H., Becker K., Matt S. and Märk T.D., Int. J. Mass Spectrom. 197, 37 (2000). doi: 10.1016/S1387-3806(99)00257-2

[28] Bethe H., Ann. Phys. 397, 325 (1930). doi: 10.1002/andp.19303970303

[29] Mulliken R.S., J. Chem. Phys. 23, 1833 (1955). doi: 10.1063/1.1740588

[30] Tang R. and Callaway J., J. Chem. Phys. 84, 6854 (1986). doi: 10.1063/1.450850

[31] Huo W.M. and Kim Y.K., Chem. Phys. Lett. 319, 576 (2000). doi: 10.1016/S0009-2614(00)00150-0

[32] Scott G.E. and Irikura K.K., Surf. Interface Anal. 37, 973 (2005). doi: 10.1002/sia.2091

[33] Scott G.E. and Irikura K.K., J. Chem. Theory Comput. 1, 1153 (2005). doi: 10.1021/ct050077j 26631658

[34] Huber S.E., Sukuba I., Urban J., Limtrakul J. and Probst M., Eur. Phys. J. D. 70, 182 (2016). doi: 10.1140/epjd/e2016-70292-4

[35] Kim Y.K., Santos J.P. and Parente F., Phys. Rev. A. 62 (14), 052710 (2000). doi: 10.1103/PhysRevA.62.052710

[36] Tao J.M., Perdew J.P., Staroverov V.N. and Scuseria G.E., Phys. Rev. Lett. 91 (4), 146401 (2003). doi: 10.1103/PhysRevLett.91.146401 14611541

[37] Staroverov V.N., Scuseria G.E., Tao J.M. and Perdew J.P., J. Chem. Phys. 119, 12129 (2003). doi: 10.1063/1.1626543

[38] Staroverov V.N., Scuseria G.E., Tao J.M. and Perdew J.P., J. Chem. Phys. 121, 11507 (2004). doi: 10.1063/1.1795692 15267588

[39] Weigend F. and Ahlrichs R., Phys. Chem. Chem. Phys. 7, 3297 (2005). doi: 10.1039/b508541a 16240044

[40] Weigend F., Phys. Chem. Chem. Phys. 8, 1057 (2006). doi: 10.1039/b515623h 16633586

[41] Stevens W.J., Basch H. and Krauss M., J. Chem. Phys. 81, 6026 (1984). doi: 10.1063/1.447604

[42] Stevens W.J., Krauss M., Basch H. and Jasien P.G., Can. J. Chem-Revue Canadienne De Chimie. 70, 612 (1992). doi: 10.1139/v92-085

[43] Cundari T.R. and Stevens W.J., J. Chem. Phys. 98, 5555 (1993). doi: 10.1063/1.464902

[44] von Niessen W., Schirmer J. and Cederbaum L.S., Comput. Phys. Rep. 1, 57 (1984). doi: 10.1016/0167-7977(84)90002-9

[45] Electron Propagator Calculations in Gaussian, https://www.auburn.edu/cosam/faculty/chemistry/ortiz/research/ept_g03.html (Accessed 7.12.2018 2018).

[46] Corzo H.H., Galano A., Dolgounitcheva O., Zakrzewski V.G. and Ortiz J.V., J. Phys. Chem. A. 119, 8813 (2015). doi: 10.1021/acs.jpca.5b00942 26226061

[47] Frisch M.J., et al., Gaussian **16** Rev. A.03 (2016).

[48] Zakrzewski V.G., Ortiz J.V., Nichols J.A., Heryadi D., Yeager D.L. and Golab J.T., Int. J. Quantum. Chem. 60, 29 (1996). doi: 10.1002/(SICI)1097-461X(1996)60:1<29::AID-QUA3>3.0.CO;2-7

[49] Deleeuw D.M., Mooyman R. and Delange C.A., Chem. Phys. Lett. 54, 231 (1978). doi: 10.1016/0009-2614(78)80090-6

[50] Rauh E.G. and Ackermann R.J., J. Chem. Phys. 70, 1004 (1979). doi: 10.1063/1.437531

[51] Sukuba I., Kaiser A., Huber S.E., Urban J. and Probst M., Eur. Phys. J. D. 70, 11 (2016). doi: 10.1140/epjd/e2015-60583-7

[52] Możejko P. and Sanche L., Radiat. Environ. Biophys. 42, 201 (2003). doi: 10.1007/s00411-003-0206-7 14523567

[53] Haque A.K.F., Sarker M.S.I., Patoary M.A.R., Shahjahan M., Hossain M.I., Uddin M.A., Basak A.K. and Saha B.C., Int. J. Quantum. Chem. 109, 1442 (2009). doi: 10.1002/qua.21980

[54] Guerra M., Parente F., Indelicato P. and Santos J.P., Int. J. Mass. Spectrom. 313, 1 (2012). doi: 10.1016/j.ijms.2011.12.003

[55] Guerra M., Parente F. and Santos J.P., Int. J. Mass. Spectrom. 348, 1 (2013). doi: 10.1016/j.ijms.2013.02.011

[56] Guerra M., Amaro P., Machado J. and Santos J.P., J. Phys. B-At. Mol. Opt. Phys. 48 (9), 185202 (2015). doi: 10.1088/0953-4075/48/18/185202

[57] Guerra M., Stohlker T., Amaro P., Machado J. and Santos J.P., J. Phys. B-At. Mol. Opt. Phys. 48 (5), 144027 (2015). doi: 10.1088/0953-4075/48/14/144027

[58] Haque A.K.F., Patoary M.A.R., Uddin M.A., Basak A.K., Saha B.C., in *Electron Correlation in Molecules – Ab Initio Beyond Gaussian Quantum Chemistry*, edited by Hoggan P., Ozdogan T. (Elsevier Academic Press Inc, San Diego, 2016). pp. 363.

[59] Kumar R. and Pal S., Rapid Commun. Mass. Spectrom. 27, 223 (2013). doi: 10.1002/rcm.6433 23239337

[60] Irikura K.K., J. Chem. Phys. 145 (9), 224102 (2016). doi: 10.1063/1.4971242 27984877

[61] Irikura K.K., J. Res. Natl. Inst. Stand. Technol. 122, 1 (2017). doi: 10.6028/jres.122.028 PMC7347380 34877123

[62] Janev R. and Reiter D., Phys. Plasmas. 11, 780 (2004). doi: 10.1063/1.1630794

[63] Huber S.E., Seebacher J., Kendl A. and Reiter D., Contrib. Plasma. Phys. 51, 931 (2011). doi: 10.1002/ctpp.201100029

